# Exploring Immune-Related Ferroptosis Genes in Thyroid Cancer: A Comprehensive Analysis

**DOI:** 10.3390/biomedicines13040903

**Published:** 2025-04-08

**Authors:** Zixuan Ru, Siwei Li, Minnan Wang, Yanan Ni, Hong Qiao

**Affiliations:** 1 Department of Endocrinology and Metabolism, The Second Affiliated Hospital of Harbin Medical University, Harbin 150086, China; clara_ru@163.com (Z.R.);; 2 Department of Breast Surgery, Harbin Medical University Cancer Hospital, Harbin 150081, China; email@siwei.li; 3 NHC Key Laboratory of Etiology and Epidemiology, Harbin Medical University, Harbin 150081, China

**Keywords:** immune-related ferroptosis, thyroid cancer, tumour microenvironment, biomarker, prognostic model, bioinformatics analysis, TCGA, qRT–PCR validation

## Abstract

**Background**: The increasing incidence and poor outcomes of recurrent thyroid cancer highlight the need for innovative therapies. Ferroptosis, a regulated cell death process linked to the tumour microenvironment (TME), offers a promising antitumour strategy. This study explored immune-related ferroptosis genes (IRFGs) in thyroid cancer to uncover novel therapeutic targets. **Methods**: CIBERSORTx and WGCNA were applied to data from TCGA-THCA to identify hub genes. A prognostic model composed of IRFGs was constructed using LASSO Cox regression. Pearson correlation was employed to analyse the relationships between IRFGs and immune features. Single-cell RNA sequencing (scRNA-seq) revealed gene expression in cell subsets, and qRT–PCR was used for validation. **Results**: Twelve IRFGs were identified through WGCNA, leading to the classification of thyroid cancer samples into three distinct subtypes. There were significant differences in patient outcomes among these subtypes. A prognostic risk score model was developed based on six key IRFGs (*ACSL5*, *HSD17B11*, *CCL5*, *NCF2*, *PSME1*, and *ACTB*), which were found to be closely associated with immune cell infiltration and immune responses within the TME. The prognostic risk score was identified as a risk factor for thyroid cancer outcomes (HR = 14.737, 95% CI = 1.95–111.65; *p* = 0.009). ScRNA-seq revealed the predominant expression of these genes in myeloid cells, with differential expression validated using qRT–PCR in thyroid tumour and normal tissues. **Conclusions**: This study integrates bulk and single-cell RNA sequencing data to identify IRFGs and construct a robust prognostic model, offering new therapeutic targets and improving prognostic evaluation for thyroid cancer patients.

## 1. Introduction

With a steadily increasing incidence worldwide [1], thyroid cancer has become one of the most common types of cancer in adolescents and young adults [2]. While surgery is the primary treatment for thyroid cancer, recurrence occurs in approximately 5–55% of patients [3,4]. Additionally, some patients eventually develop radioiodine-resistant thyroid cancer, which has a dismal prognosis, with a 10-year survival rate below 10% and a life expectancy of only 3–5 years [5,6]. The thyroid gland is known for its high immunogenicity [7,8], and the emergence of immunotherapy has accelerated the development of therapeutic agents for thyroid cancer, particularly in the treatment of advanced or metastatic stages [9]. Currently, the primary approach to immunotherapy for thyroid cancer involves immune checkpoint inhibitors [10]. However, the safety and efficacy of this treatment still require further validation. Identifying biomarkers associated with the progression of thyroid cancer is essential for discovering new therapeutic targets. These efforts are crucial for optimising diagnostic and treatment strategies and ultimately improving patient outcomes.

Ferroptosis is a unique form of cell death that depends on lipid peroxidation and iron accumulation [11]. Studies have demonstrated that ferroptosis contributes to therapy by facilitating tumour growth inhibition mediated by tumour suppressor genes [12]. Clear cell renal cell carcinoma exhibits significant ferroptosis susceptibility due to its unique metabolic characteristics [13]. MYCN-amplified neuroblastoma is highly sensitive to ferroptosis due to its excessive dependence on glutathione metabolism [14]. Notably, cancer cells harbouring RAS mutations are also prone to ferroptosis due to lipid metabolism reprogramming and iron homeostasis imbalances [15]. Based on these findings, multiple drugs have been demonstrated to exert antitumour effects by inducing ferroptosis [16,17]. Ferroptosis-related regulatory mechanisms in thyroid cancer are gradually being elucidated. NRF2, as a key antioxidant transcription factor, enhances cancer cell resistance to oxidative stress by activating downstream antioxidant proteins, thereby promoting tumour progression and increasing cell survival [18]. Additionally, the long non-coding RNA CERS6-AS1 suppresses ferroptosis by regulating the miR-497-5p/LASP1 axis, facilitating the growth and metastasis of papillary thyroid carcinoma (PTC) [19]. These findings suggest that targeting NRF2 or CERS6-AS1 may represent a promising therapeutic strategy for thyroid cancer. Although ferroptosis exhibits great potential in tumour eradication, selectively inducing tumour cell death without compromising the function of normal cells, such as immune cells, remains a significant challenge.

The concept of the tumour microenvironment (TME) was first proposed by Virchow in 1863 to reveal the complex connection between tumours and immunity [20]. In the TME, immune cells play a pivotal role and are critically involved in the ferroptosis pathway, primarily in two aspects. On the one hand, immune cells regulate the ferroptosis process in tumour cells by secreting inflammatory factors, such as IFN-γ, thereby influencing tumour growth and immune evasion [21,22]. On the other hand, the ferroptosis levels of immune cells influence their function, such as the cytotoxicity of CD8+ T cells and the polarisation state of macrophages [23,24]. Studies have shown that SCARA5 deficiency suppresses ferroptosis in tumour cells, thereby impairing immune activation within the TME and increasing the resistance of hepatocellular carcinoma (HCC) cells to sorafenib [25]. Furthermore, the downregulation of NCOA4 inhibits ferritinophagy, promoting the polarisation and function of M2 macrophages, fostering an immunosuppressive environment and accelerating tumour progression [26]. From a therapeutic perspective, combining ferroptosis inducers with a PD-1-immune checkpoint blockade has been demonstrated to significantly enhance immunotherapy efficacy [27]. Ferroptosis inducers may trigger immunogenic cell death, thereby activating antitumour immune responses within the TME and overcoming resistance to immunotherapy. These findings expand the therapeutic potential of ferroptosis in cancer treatment. Thus, a comprehensive analysis of immune-related ferroptosis genes (IRFGs) and their regulatory mechanisms within the TME holds promise for identifying novel therapeutic targets and driving the development of next-generation tumour immunotherapy strategies.

Although previous studies have demonstrated the critical role of ferroptosis in various malignancies, a comprehensive understanding of IRFGs within the TME of thyroid cancer and their impact on disease progression remains lacking. This study aimed to explore the potential roles of IRFGs in thyroid cancer. In recent years, bioinformatics analysis has been widely applied in cancer research, enabling the identification of key genes with potential clinical relevance. Leveraging data from the TCGA and GEO databases, we integrated bioinformatics approaches with validation in clinical thyroid cancer tissues to analyse the expression patterns of IRFGs in thyroid cancer and their impact on prognosis. This research is expected to establish a theoretical foundation for the development of novel therapeutic strategies in thyroid cancer.

## 2. Materials and Methods

### 2.1. Dataset Acquisition and Preprocessing

Data from TCGA-THCA were obtained using UCSC Xena. Annotation files were provided by using UCSC Xena to perform gene symbol reannotation. The clinical data of the patients corresponding to the transcriptome sequencing data, including age, sex, pathological stage and survival prognosis, were downloaded. A total of 504 thyroid cancer cases and 55 normal thyroid tissues with complete information were included.

The single-cell RNA sequencing (scRNA-seq) dataset GSE184362 was publicly available in the Gene Expression Omnibus (GEO) database. This dataset comprises 23 fresh thyroid carcinoma specimens obtained from 11 patients, including 7 primary tumours, 6 paired adjacent normal tissues, 8 lymph node metastases, and 2 subcutaneous metastases [28].

### 2.2. Estimated Immune Cell Type Fractions

The CIBERSORTx analysis tool [29] was used to calculate the relative abundance of immune cell subtypes within the TME. CIBERSORTx is a widely used computational algorithm designed to deconvolute bulk RNA sequencing data and quantify the proportions of 22 distinct immune cell subtypes, including B cells, T cells, macrophages, and dendritic cells. A reference signature matrix of gene expression profiles was utilised for this analysis, which provides a robust framework for estimating immune cell compositions across diverse tissue types. This study is based on the method proposed by Chen et al. [29], in which CIBERSORTx has been proven to reliably estimate immune cell infiltration.

To ensure the reliability of the results, the analysis was performed with 1000 permutations to minimise potential noise and random variation. This method has been applied in relevant studies [30,31]. The calculated immune infiltration score matrix served as the basis for further downstream analyses. Specifically, the immune cell fractions were used as input data for weighted gene coexpression network analysis (WGCNA), which identifies modules of highly correlated genes and investigates their relationships with immune cell infiltration patterns.

### 2.3. WGCNA

Abundance values of immune-infiltrating cells obtained from CIBERSORTx analysis were utilised to construct a coexpression network using the WGCNA (version 1.71) R package. The pickSoftTreshold function was used to determine the soft threshold. Module eigengenes were used to construct intermolecular correlations, followed by hierarchical clustering. The associations between phenotypes and gene modules were calculated using Pearson correlation, enabling the identification of modules related to specific traits and ultimately selecting the optimal module for downstream analysis.

### 2.4. Differential Expression Analysis

Ferroptosis genes were screened based on the optimal WGCNA module (Table S1). Ferroptosis genes were downloaded and merged from three databases, including the GeneCards database (546 ferroptosis-associated genes), FerrDb [32] (260 ferroptosis-associated genes), and the GSEA and MSigDB [33] databases (65 ferroptosis-associated genes) (Table S2). DESeq2 was used to analyse the expression of genes in the THCA dataset [34]. The network diagram was built via protein–protein interaction (PPI) analysis and Cytoscape (version 3.10.3) [35]. Gene Ontology (GO) enrichment analysis [36] was subsequently performed using a ClusterProfiler package (version 4.0) [37].

### 2.5. Molecular Typing

Molecular subtyping of THCA (thyroid cancer) tumour samples was performed using IRFGs identified through WGCNA. To achieve robust and reproducible clustering, we applied ConsensusClusterPlus [38], an unsupervised clustering method that iteratively resamples the data to enhance clustering stability. Spearman’s correlation was used to compute pairwise distances, as it effectively captures monotonic relationships in gene expression data. Hierarchical clustering (HC) was employed as the clustering algorithm, generating a dendrogram to illustrate the nested relationships among tumour samples [39].

The optimal number of clusters was determined based on the consensus matrix, cumulative distribution function (CDF) curves, and delta area plots, ensuring stable and reliable classification [40]. The prognostic significance of the identified molecular subtypes was evaluated, and differences in overall survival (OS) across subtypes were statistically assessed using the log-rank test. This analysis revealed distinct survival profiles among subtypes, emphasising the clinical relevance of IRFG-based molecular subtyping in THCA and its potential prognostic value.

### 2.6. Construction of the Prognostic Model

To develop a robust prognostic model, tumour samples were split 7:3 between the training and validation sets at random. Specifically, the training set comprised 353 samples, whereas the validation set included 151 samples. This stratification ensured sufficient data for model training while retaining an independent dataset for performance evaluation. Similar experimental methods have been used in previous studies [41,42].

Least absolute shrinkage and selection operator (LASSO) regression was performed on the training dataset to identify the variables most strongly associated with OS. LASSO regression employs L1 regularisation, which shrinks some regression coefficients to zero, effectively selecting the most prognostically relevant features while preventing overfitting [43,44]. By integrating Cox proportional-hazards regression, LASSO-Cox regression enables the selection of key prognostic genes and determines their regression coefficients for constructing a risk prediction model [45]. In this study, we used LASSO-Cox regression to select six IRFGs, which were incorporated into the prognostic risk model. The risk score was determined as a weighted linear combination of the selected variables, with weights corresponding to the respective regression coefficients of thyroid cancer patients. The median risk score was used as a cutoff to stratify the patients into two distinct subgroups. This dichotomous classification aligns with clinical decision-making processes.

To assess the clinical relevance and performance of the risk model, Kaplan–Meier (KM) survival analysis was conducted to compare OS between the high-risk and low-risk groups [46]. The log-rank test was used to statistically evaluate survival differences. The results demonstrated that patients in the high-risk group exhibited significantly worse OS, highlighting the potential of this model for risk stratification and prognostic assessment in thyroid cancer.

To further evaluate the predictive power of the model, we performed time-dependent receiver operating characteristic (TimeROC) analysis [47], which estimates the area under the curve (AUC) at different time points. A higher AUC value indicates better discriminative ability of the model in predicting patient outcomes. The results confirmed that the model exhibited strong predictive performance in both the training and validation cohorts, supporting its robustness for long-term survival prediction.

### 2.7. Mutation Analysis

Somatic mutation data and microsatellite instability (MSI) profiles were obtained from the publicly available TCGA-THCA dataset. These data provide a comprehensive overview of the genetic alterations in thyroid carcinoma, enabling detailed exploration of mutation patterns and their potential clinical significance.

Mutation data were analysed using the Maftools [48] R package (version 2.10.05), a widely used tool for visualising and summarising mutation annotations. Key metrics such as tumour mutational burden (TMB), frequently mutated genes, and mutation types (e.g., single nucleotide variants, insertions, and deletions) were calculated for all samples. Comparisons between different molecular subtypes identified through clustering were performed to explore associations between mutation patterns and tumour characteristics. Fisher’s exact test was employed to determine significant differences in mutation frequencies between groups.

Additionally, microsatellite instability (MSI) status was evaluated as a genomic marker of interest because of its implications in immunotherapy response. The MSI-high and -low groups were compared to investigate potential correlations with immune-related ferroptosis gene expression, immune infiltration, and OS.

### 2.8. Construction of the Clinical Prediction Model

Univariate Cox regression analysis was employed to assess the prognostic significance of clinicopathological features combined with the risk score in predicting outcomes. The risk score and clinical character were integrated to build a model, resulting in the development of a clinical prediction nomogram for risk assessment. Harrell’s concordance index (C-index) was used to evaluate the model’s ability to distinguish between high-risk and low-risk patients effectively, while calibration curves were employed to evaluate the consistency between the prediction and the observed outcomes of thyroid patients.

### 2.9. GSVA Analysis

Rank statistics were calculated to determine the associations between genes and specific features. For each gene set across individual samples, enrichment scores were obtained from Gene set variation analysis (GSVA), resulting in the generation of an enrichment score matrix [49]. The immune gene set [50] was retrieved from the ImmPort database, comprising 2483 immune response-related genes, which were downloaded for analysis (Table S3).

### 2.10. Quality Control of Single-Cell Data

The single-cell dataset was analysed using the Seurat package (version 4.0) in R [51]. Seurat was used to import the resulting array of 23 samples in the single cell sequencing data and create the Seurat object for this analysis. The cells were filtered following the original text. According to the quality control standards in the original text (nFeature_RNA < 500, nFeature_RNA > 5000), low-quality cells were removed, and the doubletFinder_v3 function was used to eliminate the influence of twins.

The data were normalised using the log normalisation method using the NormalizeData function, which also accounted for sequencing depth to ensure consistency across samples. After controlling for mean expression and dispersion relationships, highly variable genes (HVGs) were identified as these genes likely capture biologically relevant differences. RunPCA was employed to perform principal component analysis (PCA) on the expression data of these HVGs, generating a set of principal components ranked by explained variance. The number of significant principal components was determined using ElbowPlot, and these components were subsequently used for further analysis. To show the data in a reduced-dimensional space, the top 15 statistically significant principal components were chosen as inputs for t-distributed stochastic neighbour embedding (t-SNE).

### 2.11. Cluster Analysis and Annotation

The FindClusters function was utilised for cell clustering and subsequent cell type identification. The marker genes used in the cell type annotations were cross-referenced with the original literature to confirm the accuracy of the cell type assignments. Myeloid cells were identified on the basis of the expression of markers such as LYZ, FCER1G, TYROBP, S100A8, S100A9, and CD14; T/NK cells by CD3D, CD3E, CD3G, CD247, IL7R and IL32; and B cells by CD79A, CD79B, MS4A1, IGKC, CD74, and IGHD. Thyrocytes were identified via markers, including TG, CLU, FN1, MGST1, S100A13, EPCAM, KRT18, and KRT19. Fibroblasts are characterised by ACTA2, COL1A1, COL1A2, COL3A1, IGFBP7, RGS5, and TAGLN, and endothelial cells are characterised by CDH5, CD34, CLDN5, MGP, PECAM1, RAMP2, TFPI, TIMP3, and VWF. Differential marker genes among the identified cell groups were further analysed.

The Louvain algorithm was used via the FindClusters function to optimise clustering. The FindAllMarkers function was used to recognise marker genes for each cluster by applying the Wilcoxon rank-sum test, which compares gene expression levels between a specific cluster and all other clusters.

### 2.12. Cel Communication

The cell communication network was analysed using the CellChat R package (version 1.1.3) [52], a robust computational tool designed to infer and visualise intercellular communication on the basis of single-cell or bulk transcriptomic data. This approach allows a comprehensive understanding of how different cell populations within the tumour microenvironment interact through ligand–receptor signalling pathways.

To illustrate the interaction network, a circle diagram was generated, which shows the intensity and directionality of signalling exchanges among various cell populations. The diagram provides a global view of the communication landscape, highlighting key signalling pathways and their contributions to the functional dynamics of the TME. Furthermore, the analysis included a bubble plot to quantify and visualise the key ligand–receptor pairs that mediate intercellular communication. The size and colour represent the strength and significance of specific interactions, offering detailed insights into their roles in signal transmission.

### 2.13. RNA Sequencing (RNA-Seq) Analysis

The RNA sequencing (RNA-seq) data used for validation were derived from prior sequencing results obtained by our research group. The dataset included thyroid tumour tissues (*n* = 3) and their corresponding adjacent normal thyroid tissues (*n* = 3), providing a paired analysis framework to investigate tumour-specific transcriptomic changes [53].

### 2.14. Quantitative Real-Time PCR (qRT–PCR)

#### 2.14.1. Clinical Samples

Thyroid cancer patients who visited the 2nd Affiliated Hospital of Harbin Medical University between March and December 2022 were recruited for this study. The inclusion criteria included a confirmed histopathological diagnosis of thyroid cancer and the availability of paired tumour and adjacent normal tissues. Tumour and adjacent normal tissues were immediately collected during surgical resection and preserved in liquid nitrogen to maintain RNA, DNA, and protein integrity for downstream analyses. Clinical information, including demographic data, pathological stage, and follow-up outcomes, was also collected. The Ethics Committee of the 2nd Affiliated Hospital of Harbin Medical University approved the study (Approval No. KY2020-022) on 20 January 2020, ensuring adherence to ethical standards and patient confidentiality. All procedures complied with the Declaration of Helsinki and relevant national guidelines for human research.

#### 2.14.2. qRT–PCR

Total RNA was extracted from tissue samples using a TRIzol reagent (Invitrogen, Carlsbad, CA, USA), following the manufacturer’s protocol. The purity and concentration of the extracted RNA were assessed using a NanoDrop spectrophotometer (Invitrogen, Carlsbad, CA, USA), ensuring a 260/280 ratio between 1.8 and 2.0 for downstream analysis. The RNA integrity was further verified using agarose gel electrophoresis. For cDNA synthesis, reverse transcription was performed using a reverse transcription kit (RR047A, Takara, Kusatsu, Japan). Quantitative real-time PCR (qRT–PCR) was conducted on a QuantStudio 5 real-time PCR system (Applied Biosystems, Foster City, CA, USA) using the SYBR Green-based detection method. This method has been applied in relevant studies [54,55]. The relative gene expression levels were statistically analysed using paired t-tests. GAPDH was used as an internal control. The sequences of the primers used in this study are listed in Table S4.

### 2.15. Statistical Methods

All data calculations and statistical analyses were performed using R programming (version 4.1). The statistical analysis methods were integrated under the relevant headings.

## 3. Results

**Figure 1:**
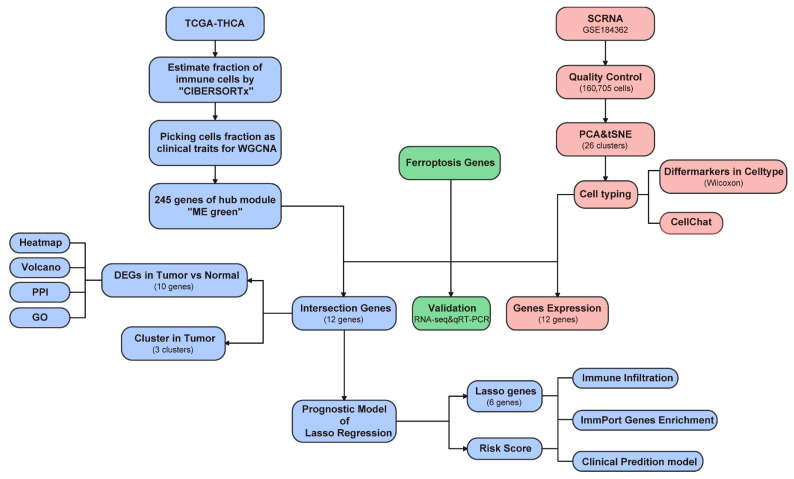
Overall design flow chart.

The study design is illustrated in Figure 1.

### 3.1. Identification of Immune-Related Modules Using WGCNA

**Figure 2:**
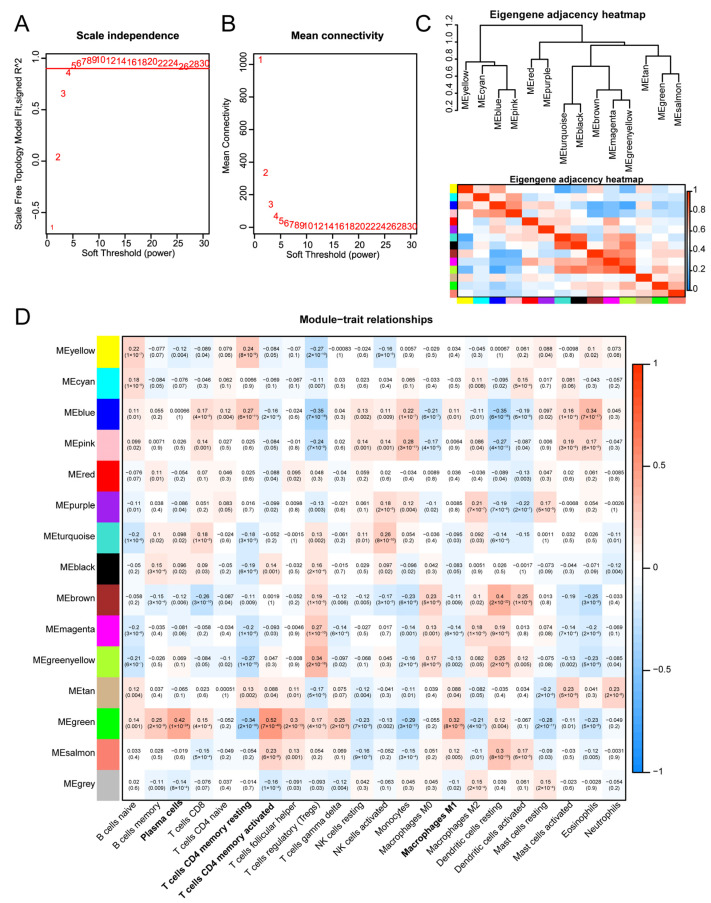
Identification of key modules: (**A**) Scale-free fit index for the soft threshold power (β) in analyses 1–30. (**B**) Examination of the average connectivity across the 1–30 soft threshold powers (β). (**C**) Correlation map between 14 important modules. (**D**) Association diagram of 14 modules and phenotypes. The MEgreen module showed the highest correlation with immune cells, including activated memory CD4+ T cells, with a correlation coefficient of 0.52 and *p* = 7 × 10^−40^.

CIBERSORTx was used to estimate the abundance of immune cell infiltration in the TCGA-THCA data, and the abundance values of 22 immune cells were obtained as trait data for WGCNA, which was employed to construct a coexpression network. First, we calculated a soft threshold β, which resulted in a value of 4, thus ensuring that the network we constructed was in line with the characteristics of the scale-free network (Figure 2A,B). In this study, we identified 14 modules for subsequent analysis, and the modules were visualised using an intermodule correlation heatmap (Figure 2C). Among these 14 modules, MEgreen was identified as the hub module. As shown in Figure 2D as bold text, the MEgreen module was correlated with various kinds of infiltrating immune cells, including activated memory CD4+ T cells (R = 0.52, *p* = 7 × 10^−40^), plasma cells (R = 0.42, *p* = 1 × 10^−24^), resting memory CD4+ T cells (R = −0.34, *p* = 2 × 10^−16^), and M1 macrophages (R = 0.32, *p* = 8 × 10^−15^). This module consisted of 245 genes, including *ALOX5AP*, *ACSL5*, *HMOX1M*, *STAT1*, *STAT2*, *CYBB*, and *SOD2* (Table S5).

### 3.2. Expression Analysis of IRFGs

**Figure 3:**
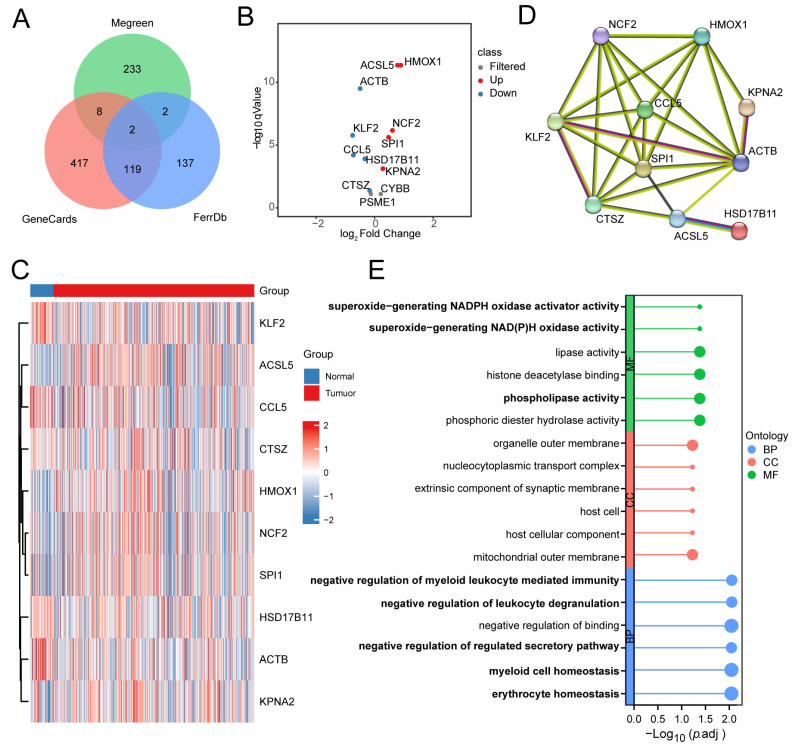
Differential expression analysis of immune-related ferroptosis genes (IRFGs): (**A**) Identification of IRFGs. (**B**,**C**) The expression of 12 IRFGs in thyroid tumour compared with normal tissues. The expression of *ACTB*, *KLF2*, *CCL5*, *HSD17B11*, and *CTSZ* was downregulated. The expression of *ACSL5*, *HMOX1*, *NCF2*, *SPI1*, and *KPNA2* was upregulated. (|log2FC| > 0 and FDR < 0.05). (**D**) PPI network diagram illustrating the 10 IRFGs. (**E**) Gene Ontology (GO) analysis of the differentially expressed ferroptosis genes. The bold text represents the potential biological processes involved.

Next, we intersected the gene list from this module in our study with ferroptosis gene sets from three public datasets. Through this process, we identified a total of 12 IRFGs (Figure 3A), including *ACSL5*, *ACTB*, *KLF2*, *CCL5*, *HSD17B11*, *CTSZ*, *KPNA2*, *SPI1*, *NCF2*, *HMOX1*, *PSME1*, and *CYBB*, which are associated with both immune regulation and ferroptosis mechanisms and may play critical roles in disease onset and progression.

On the basis of the 12 IRFGs in the MEgreen module, we first explored the differences in expression between the thyroid tumour and normal groups via the TCGA-THCA dataset. The genes with significant differences were screened according to the threshold (|log2FC| > 0, FDR < 0.05), and 10 ferroptosis genes exhibited differential expression, including *ACSL5*, *ACTB*, *KLF2*, *CCL5*, *HSD17B11*, *CTSZ*, *KPNA2*, *SPI1*, *NCF2*, and *HMOX1* (Table S6, Figure 3B,C). A protein–protein interaction network was constructed, and the diagram was subsequently generated, identifying *ACTB*, *CCL5*, *KPNA2*, *SPI1*, *NCF2*, and *HMOX1* as hub proteins in ferroptosis-related biological processes (Figure 3D).

ClusterProfiler was utilised to perform an enrichment analysis of the differences in ferroptosis genes between groups to further investigate potential biological processes (Table S7). GO analysis indicated that the differentially expressed ferroptosis genes were mainly involved mainly in molecular functions such as superoxide-generating NADPH oxidase activator activity, superoxide-generating NAD(P)H oxidase activity and phospholipase activity. The enriched biological processes included the negative regulation of leukocyte degranulation, negative regulation of myeloid leukocyte mediated immunity, negative regulation of regulated secretory pathway, myeloid cell homeostasis, and erythrocyte homeostasis (Figure 3E).

### 3.3. Molecular Typing of the THCA Tumour Samples

**Figure 4:**
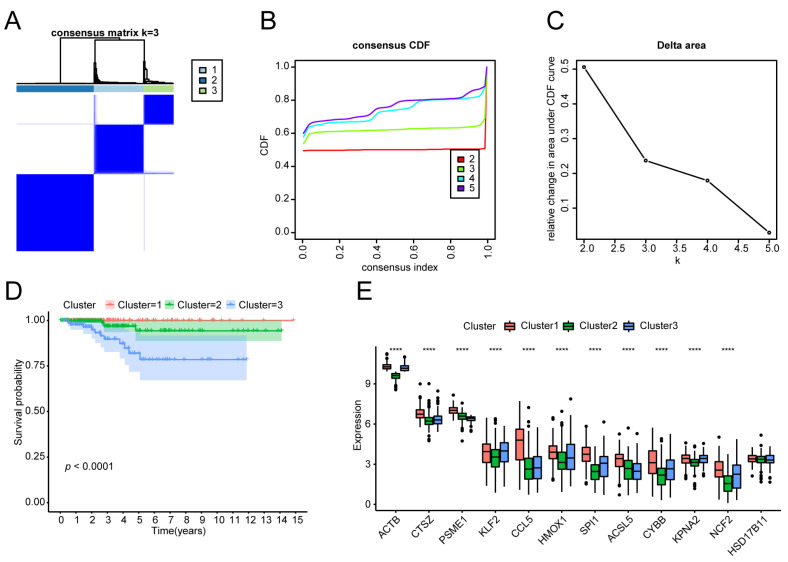
Tumour subtype identification and Kaplan–Meier (K–M) analysis: (**A**) The consensus clustering matrix heatmap illustrates the clustering stability, represented by a gradient from white to dark blue. (**B**) The cumulative distribution function (CDF) curve was generated to evaluate overall changes in clustering stability, aiding in the assessment of additional clusters. (**C**) Delta area curve. The elbow point on the delta area curve, where the rate of change markedly decreases, typically corresponds to the optimal number of clusters. (**D**) Survival curves of the different subtype groups, *p* < 0.0001. (**E**) The expression of the 12 immune-associated ferroptosis genes differed among the thyroid tumour subtypes, as shown in the box plot. ****: *p* < 0.0001. •: outliers.

Based on the expression of the 12 ferroptosis genes in the MEgreen module and the FPKM expression level data of the thyroid cancer tumour samples, we used the ConsensusClusterPlus consistent clustering method to classify the thyroid cancer tumour samples. Through a comprehensive analysis of the consistency matrix heatmap, CDF, and delta area curve, we determined that a clustering number of three provided the most stable classification and optimal differentiation of the samples (Figure 4A–C). We further performed K–M analysis on the three subtypes. The OS of patients with various tumour subtypes was significantly different (*p* < 0.0001, Figure 4D). The expression of 12 ferroptosis module genes differed among the tumour subtypes. In particular, the expression levels of *ACTB*, *CTSZ*, *PSME1*, *KLF2*, *CCL5*, *HMOX1*, *SPI1*, *ACSL5*, *CYBB*, *KPNA2*, and *NCF2* were greater among the three subtypes (Figure 4E).

### 3.4. Construction of the Prognostic Gene Model

**Figure 5:**
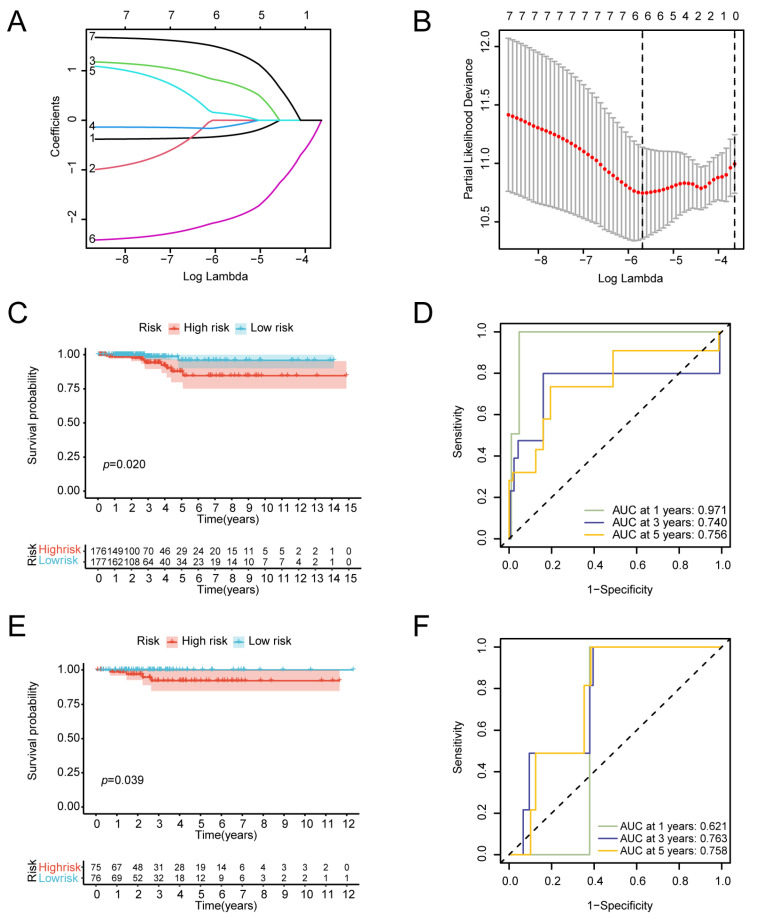
Correlations between IRFGs and the prognosis of thyroid cancer patients: (**A**) LASSO analysis was used to construct a fitting model, with a plot illustrating the variation in lambda values for the seven ferroptosis genes significantly associated with prognosis, including *ACTB*, *ACSL5*, *CYBB*, *HSD17B11*, *NCF2*, and *PSME1*. (**B**) Cross-validation analysis was conducted to assess the optimal regularisation parameter. This approach allows the construction of a model that effectively balances avoiding overfitting while maintaining strong predictive performance, thereby optimising the model’s overall performance. A risk model consisting of six genes (*ACSL5*, *HSD17B11*, *CCL5*, *NCF2*, *PSME1*, and *ACTB*) was constructed. (**C**) A survival difference between the two groups in the training set was revealed through K–M analysis (*p* = 0.02). (**D**) Time-dependent ROC curve for the training set. (**E**) A survival difference between groups in the validation set was revealed through K–M analysis (*p* = 0.039). (**F**) Time-dependent ROC curve for the validation set.

To determine the association between the 12 IRFGs and the prognosis in patients with thyroid cancer, we analysed TCGA-THCA sequencing data along with survival information. Survival analysis of these genes in tumour samples identified seven genes that were associated with prognosis, including *ACSL5*, *HSD17B11*, *CCL5*, *NCF2*, *PSME1*, *ACTB*, and *CYBB* (Table S8). The TCGA-THCA tumour samples were randomly divided into two groups at a ratio of 7:3. One group was used as the training set to develop a prognostic model, and the other group was utilised as the validation set. We used LASSO Cox regression to construct a model for the evaluation of thyroid cancer prognosis (Figure 5A,B), and a risk model consisting of six genes (*ACSL5*, *HSD17B11*, *CCL5*, *NCF2*, *PSME1*, and *ACTB*) was subsequently developed. The risk score for thyroid cancer patients was determined using the following formula:

(1) Risk Score=−(0.3062202×ACSL5)+(0.7545444×HSD17B11)−(0.1150395×CCL5)+(0.1253110×NCF2)−(1.9902819×PSME1)+(1.4114612×ACTB).

Patients were categorised into low-risk and high-risk groups based on the median risk score. K–M analysis revealed a significant difference in survival between the two groups in the training set (*p* = 0.02) (Figure 5C). The validation set showed a difference in OS between the two groups as well (*p* = 0.039) (Figure 5E), further supporting the predictive capability of the model. We also conducted a TimeROC curve analysis to demonstrate the predictive performance for OS. In the training set, the areas under the curve (AUCs) for 1-year, 3-year, and 5-year OS were 0.971, 0.740, and 0.756. In the validation set, the AUCs for the 1-year, 3-year and 5-year OS rates were 0.621, 0.763, and 0.758 (Figure 5D,F).

### 3.5. Immune Signature Correlation Analysis

**Figure 6:**
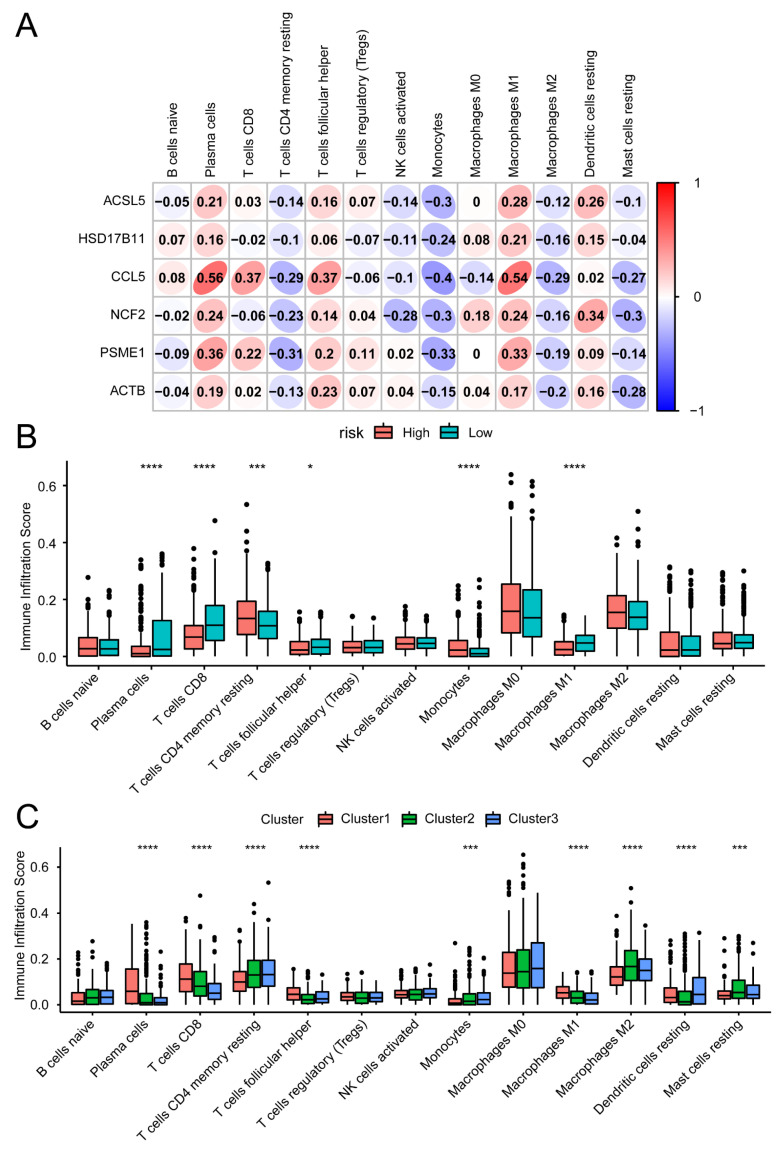
Correlation analysis of immune infiltration: (**A**) In the microarray expression data, the prognostic ferroptosis genes *ACSL5*, *HSD17B11*, *CCL5*, *NCF2*, *PSME1*, and *ACTB* were correlated with immune infiltration, as shown in the heatmap. (**B**) Immune cell infiltration scores were assessed for both the high- and low-risk groups. Low-risk samples presented increased CD8+ T cells and M1 macrophage immune infiltration scores. High-risk samples presented increased CD4+ memory T cell and monocyte immune infiltration scores. (**C**) Comparison of infiltration levels of 13 immune Cell types across three tumour subtypes. *: *p*< 0.05, ***: *p* < 0.001, ****: *p* < 0.0001. •: outliers.

We investigated the relationships between IRFGs and TME. The CIBERSORTx online tool was utilised to assess immune cell infiltration on the basis of microarray expression profiling data, and the abundances of 22 types of immune cells were estimated. Pearson correlation analysis revealed the relationships between the six prognostic genes and immune cell populations (Table S9, Figure 6A). Specifically, monocyte abundance was correlated with *ACSL5* (R = −0.3), plasma cell abundance was correlated with *CCL5* (R = 0.56), and M1 macrophage abundance was correlated with *CCL5* (R = 0.54). Resting dendritic cells were correlated with *NCF2* (R = 0.34), plasma cell abundance was correlated with *PSME1* (R = 0.36), and mast cell abundance was correlated with *ACTB* (R = −0.28). In addition, the infiltration scores of immune cells were calculated for both the high- and low-risk groups (Figure 6B). Notably, the infiltration scores for plasma cells, CD8+ T cells, and M1 macrophages were higher in the low-risk samples. The infiltration scores for CD4+ memory T cells and monocytes were greater in the high-risk samples. Similarly, the immune infiltration scores differed among the tumour subtypes (Figure 6C).

**Figure 7:**
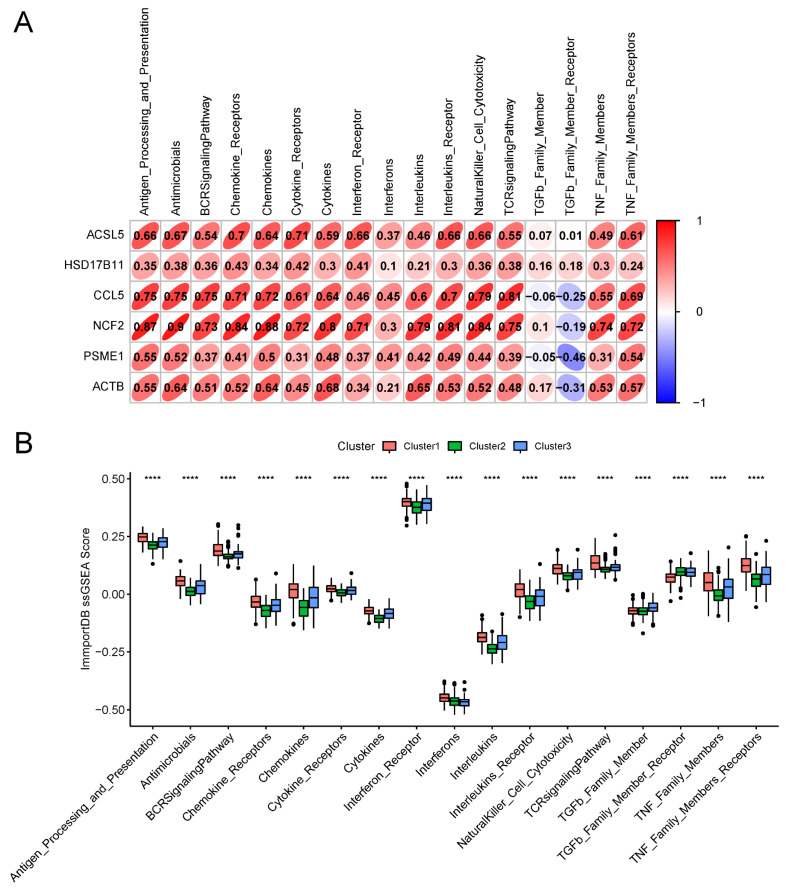
Correlation analysis of immune response features: (**A**) Pearson correlation analysis indicated a relationship between the six prognostic genes and immune responses in thyroid cancer, as depicted in the heatmap. (**B**) Differential analysis of the 17 different immune response scores among the three tumour subtypes. ****: *p* < 0.0001. •: outliers.

Furthermore, we analysed the immune response in thyroid cancer. The relative enrichment scores of 17 immune responses in thyroid cancer patients were calculated using ssGSEA from the GSVA (version 1.42.0) R package. Pearson correlation analysis was performed to reveal the relationships between the six genes in the prognostic model and immune response activity (Table S10, Figure 7A). The activity of cytokine receptors was positively correlated with *ACSL5* (R = 0.71), the activity of the TCR signalling pathway was positively correlated with *CCL5* (R = 0.81), the activity of antimicrobials was positively correlated with *NCF2* (R = 0.9), the activity of antigen processing and presentation was positively correlated with *PSME1* (R = 0.55), and there was a positive correlation between cytokine activity and *ACTB* (R = 0.68). In addition, we used a box plot to observe the differences in each immune response among the three tumour subtypes (Figure 7B), and all immune responses were significantly different among the tumour subtypes.

### 3.6. Effect of the Risk Score on Genomic Alterations

**Figure 8:**
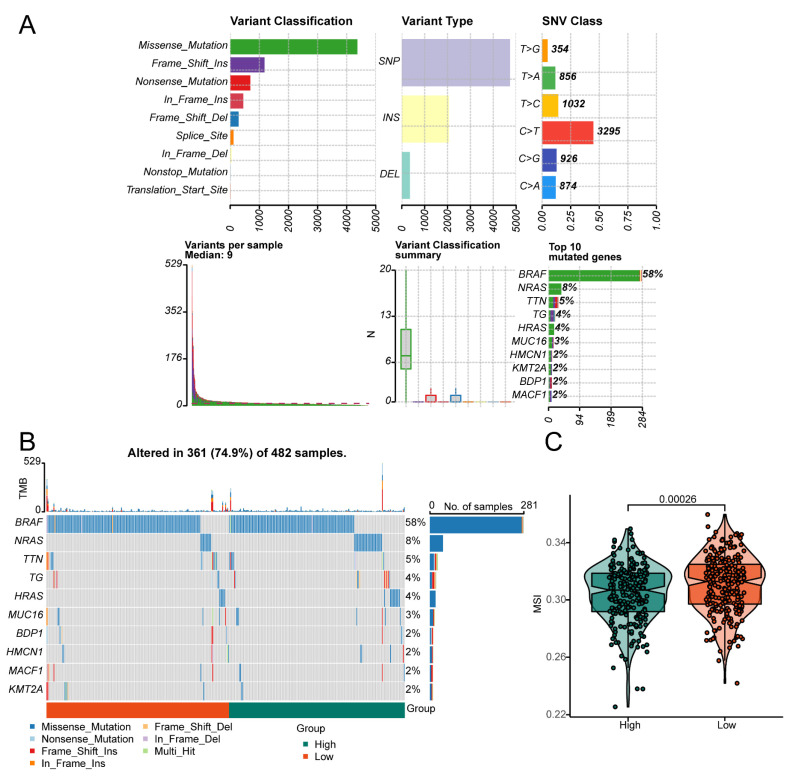
Effect of the risk score on genomic alterations: (**A**) Overall mutations of THCA. (**B**) Mutation map of the common tumour driver genes among the high-risk and low-risk patients. Each sample’s mutation information for each gene is presented, with different colors representing various mutation types. (**C**) The high-risk group demonstrated a decreased level of microsatellite instability (MSI-L) compared to the low-risk group (*p* = 0.00026).

In order to assess how the risk score affects genetic changes, we downloaded the somatic mutation data of thyroid cancer patients and used Maftools to visualise the overall situation of the somatic mutations (Figure 8A). A mutation waterfall map showed the top 10 mutated genes in thyroid cancer, including *BRAF*, *NRAS*, *TTN*, *TG*, *HRAS*, *MUC16*, *BDP1*, *HMCN1*, *MACF1*, and *KMT2A*. The results showed that the *BRAF* mutation is the most prevalent driver mutation, with a mutation rate of 58%. No significant differences were observed in the highly mutated genes between patients classified as high-risk and low-risk groups (Figure 8B). Studies have shown that the *BRAF V600E* mutation can influence the tumour immune microenvironment by activating the MAPK pathway [56] and may inhibit ferroptosis [57], suggesting its potential role in immune evasion and treatment resistance. Therefore, future research could further explore whether *BRAF V600E* inhibitors, such as Vemurafenib and Dabrafenib, cooperate with IRFGs to regulate the ferroptosis pathway, thereby influencing tumour progression and immune therapy response. Microsatellite instability (MSI) is a form of genomic instability caused by defects in the DNA mismatch repair (MMR) system [29]. We downloaded the MSI data of patients with thyroid cancer. The high-risk group demonstrated a decreased level of microsatellite instability (MSI-L) compared to the low-risk group (*p* = 0.00026, Figure 8C), indicating a worse prognosis and greater aggressiveness.

### 3.7. Construction of the Predictive Nomogram Model

**Figure 9:**
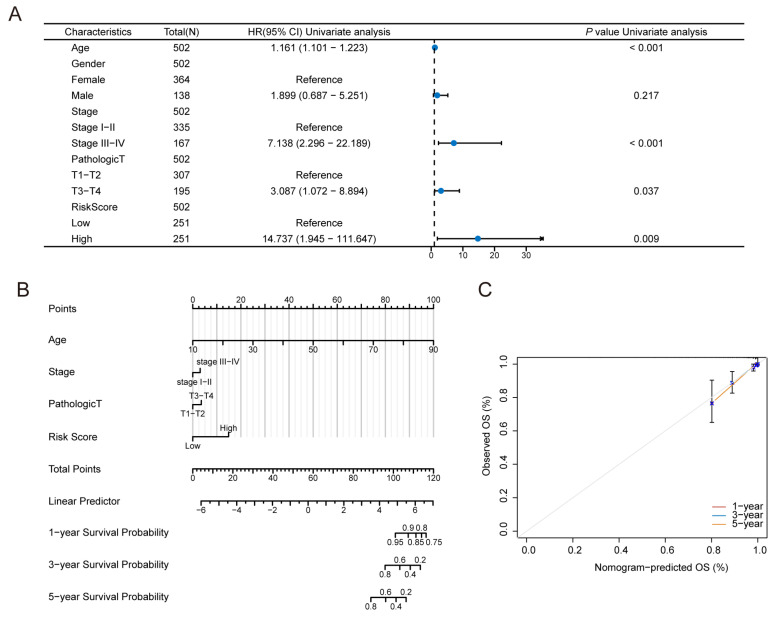
Construction of the predictive nomogram model: (**A**) Relationships between survival outcomes and clinical characteristics were assessed using univariate Cox regression analysis. The risk score was a risk factor affecting overall survival in thyroid cancer patients (HR 14.737, 95% CI 1.945–111.647; *p* = 0.009). (**B**) A nomogram was constructed based on the ferroptosis prognostic model, incorporating age, clinical stage, pathological stage T, and the risk score into the predictive model. (**C**) The C-index revealed a high degree of discrimination of the nomogram (concordance = 0.944228).

We subsequently evaluated the effects of the risk score and different clinicopathological characteristics of thyroid cancer patients. Univariate Cox regression revealed that age (HR 1.61, 95% CI 1.101–1.223, *p* < 0.001), tumour stage (HR 7.138, 95% CI 2.296–22.189, *p* < 0.001), pathological stage (HR 3.087, 95% CI 1.072–8.894, *p* = 0.037), and ferroptosis risk score (HR 14.737, 95% CI 1.945–111.647, *p* = 0.009) were risk factors for thyroid cancer (Figure 9A). A predictive nomogram was subsequently constructed to predict OS (Figure 9B). The C-index revealed a high degree of discrimination of the nomogram (concordance = 0.944228). The calibration curve revealed that when the nomogram was compared, the 1-, 3-, and 5-year OS evaluations and the actual observed values of the patients were consistent (Figure 9C).

### 3.8. Single-Cell Data Revealed the Cellular Heterogeneity of Thyroid Cancer Cells

**Figure 10:**
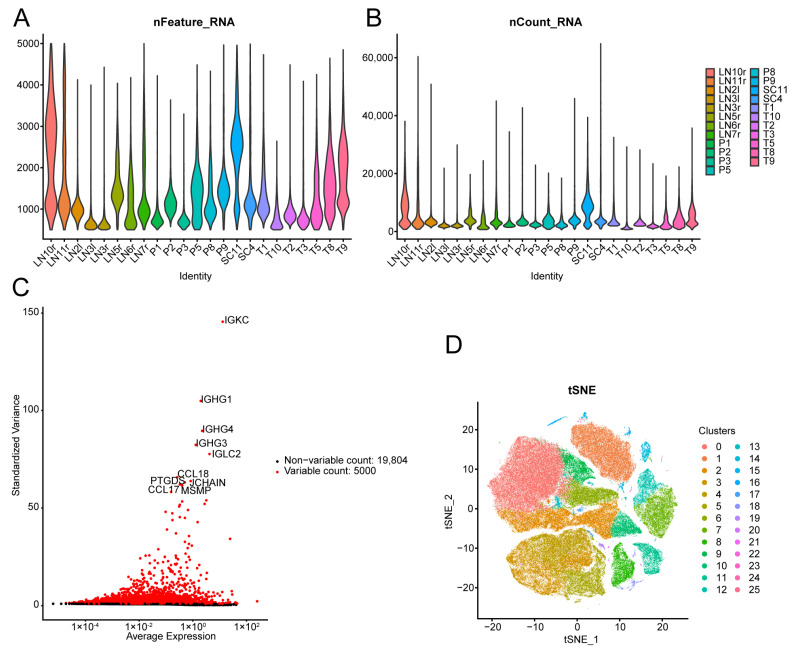
Cell quality control and dimensionality reduction cluster analysis of single-cell sequencing (scRNA-seq) data: (**A**) ScRNA-seq was performed on 23 samples, and the number of genes detected in each sample is visualised via a violin plot. (**B**) A violin plot of UMI counts was used to evaluate and compare the transcript capture depth across individual cells. (**C**) Scatter plot of the standard deviation showing the hypervariable genes present in the cells, with the top 10 genes displayed. (**D**) Dimensionality reduction cluster analysis and t-distributed stochastic neighbour embedding (t-SNE) of the cell clusters. Different colours mark the different clusters.

ScRNA-seq was performed on 23 samples. After filtering according to the quality control standards of the original text and eliminating the effect of the lot, a total of 160,705 cells were obtained (Figure 10A,B). After the normalisation of the data, the top 10,000 hypervariable genes were selected and subjected to extracting for PCA dimensionality reduction, and the top 10 genes were identified (Figure 10C). We used the SNN algorithm for cluster analysis and t-SNE dimensionality reduction to visualise single-cell clusters, and 26 optimal cell clusters were ultimately obtained (Figure 10D).

**Figure 11:**
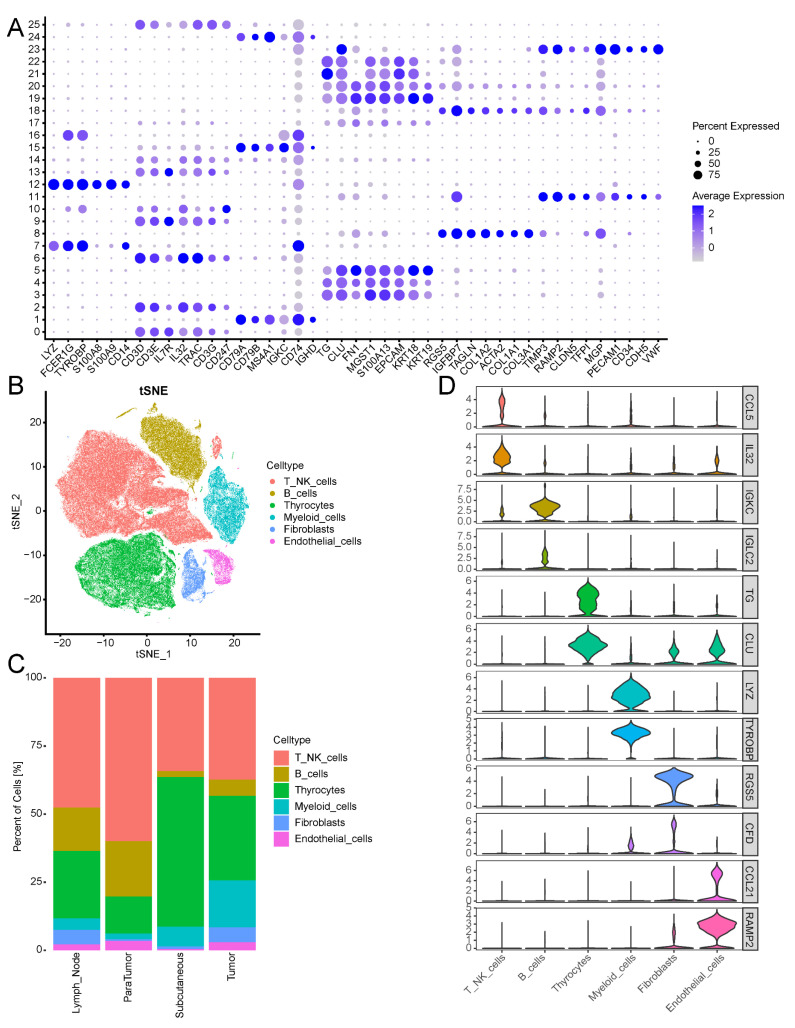
Characterisation of the scRNA-seq data: (**A**) The expression patterns of known marker genes in each cell cluster are visualised using a bubble plot. The proportion of gene expression is represented by the size of the bubbles within each cluster, while deeper colors indicate higher average expression levels. (**B**) Distribution of t-SNEs in different cell types. (**C**) A stacked column graph was generated to observe the proportions of cell types in different samples. (**D**) Violin plot showing the top 2 DEGs in each cell type.

We visualised the expression patterns of markers from previously published studies within the selected dataset using bubble plots (Figure 11A). On the basis of this analysis, the cell types were identified (Figure 11B). A total of 76,226 cells from Clusters 0, 2, 6, 9, 10, 13, and 14 were annotated as T/NK cells, accounting for 47.4% of the total cells. Clusters 3, 4, 5, 17, 19, 20, 21, and 22 were identified as thyrocytes (thyroid cells), comprising 40,279 cells (25.1%). Clusters 1, 15, 16, and 24 were identified as B cells, totalling 21,699 cells (13.5%). The myeloid cells were grouped into Clusters 7 and 12 (12,177 cells; 7.6%). Clusters 8 and 18 were annotated as fibroblasts (6034 cells; 3.7%), whereas Clusters 11 and 23 were identified as endothelial cells (4290 cells; 2.7%). To observe the proportions of cell types in the samples from different sources, we generated a stacked column graph for visualisation (Figure 11C). T/NKs and thyrocytes accounted for relatively high proportions of the total cells in the samples. Subsequently, differential expression analysis between the cell groups was performed (Table S11), and the top two marker genes in each cell type were visualised via violin plots (Figure 11D).

### 3.9. Cell Communication

**Figure 12:**
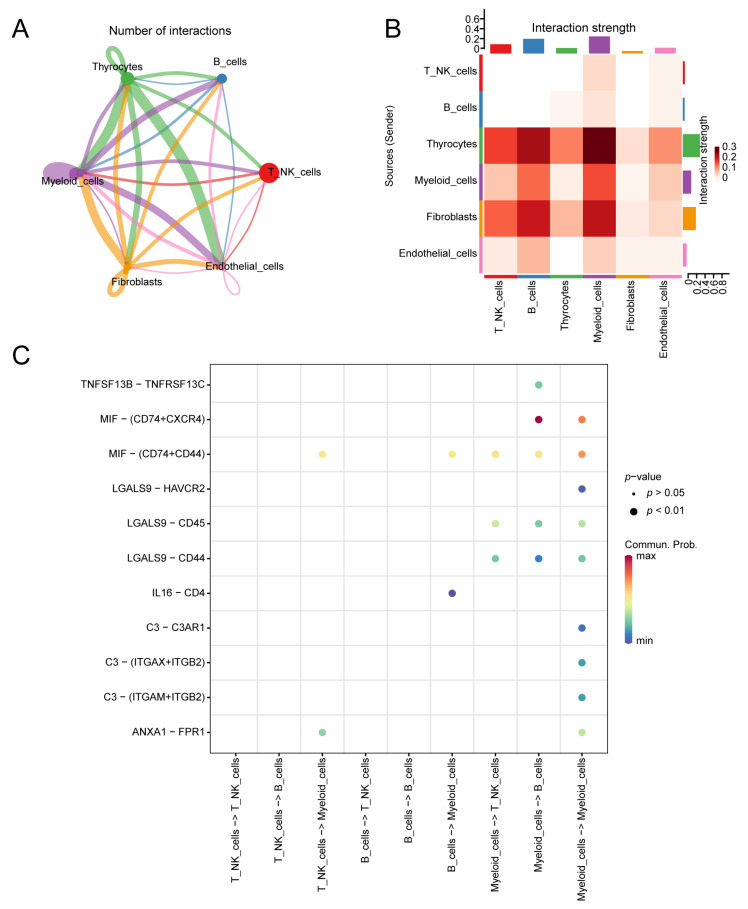
Analysis of cell communication: (**A**) The network diagram of intercellular interactions displays the quantity of interaction pairs. The thickness of the connecting lines signifies the number of significant ligand–receptor interaction pairs detected between cell types, with thicker lines indicating more interaction pairs. (**B**) The heatmap of intercellular interaction strength shows the intensity of ligand–receptor interactions, with redder colors indicating stronger interaction intensity. Among them, thyrocytes, as ligand cells (weight sum = 0.919), and myeloid cells, as receptor cells (weight sum = 0.752), presented the highest combined communication intensity. (**C**) The ligand–receptor pairs representing intercellular communication within cell groups are illustrated. The columns represent the interacting cell types, the circle size indicates the significance level, and redder circles represent higher communication probabilities between interacting cells. The communication probability for the MIF-(CD74+CXCR4) interaction between myeloid cells and B cells was 0.080.

CellChat was utilised to investigate cell communication. By integrating gene expression data from single-cell RNA sequencing with CellChat’s curated database, we inferred cell state-specific signalling pathways and communication networks. The interaction network and heatmap were used to visualise interactions and signal strength between cell types. From the network diagram, we observed a substantial number of ligand–receptor pairs, including eight pairs between thyrocytes as ligand cells and myeloid cells as receptor cells, nine pairs between thyrocytes and endothelial cells as receptor cells, nine pairs between myeloid cells as both ligand and receptor cells, and seven pairs between fibroblasts as ligand cells and myeloid cells as receptor cells (Figure 12A, Table S12). The interaction strength heatmap illustrates the communication intensity between cell types. Among them, thyrocytes, as ligand cells (weight sum = 0.919), and myeloid cells, as receptor cells (weight sum = 0.752), presented the highest combined communication intensity. This finding indicates stronger interactions between these cell types and others (Figure 12B, Table S13). Figure 12C shows the ligand–receptor pairs involved in intercellular communication within immune cell populations. The communication probability for the MIF-(CD74+CXCR4) interaction between myeloid cells and B cells was 0.080, whereas the probabilities for the MIF-(CD74+CXCR4) and MIF-(CD74+CD44) interactions within myeloid cells were 0.0510 and 0.047, respectively (Table S14).

### 3.10. Expression of IRFGs in Single-Cell Datasets

**Figure 13:**
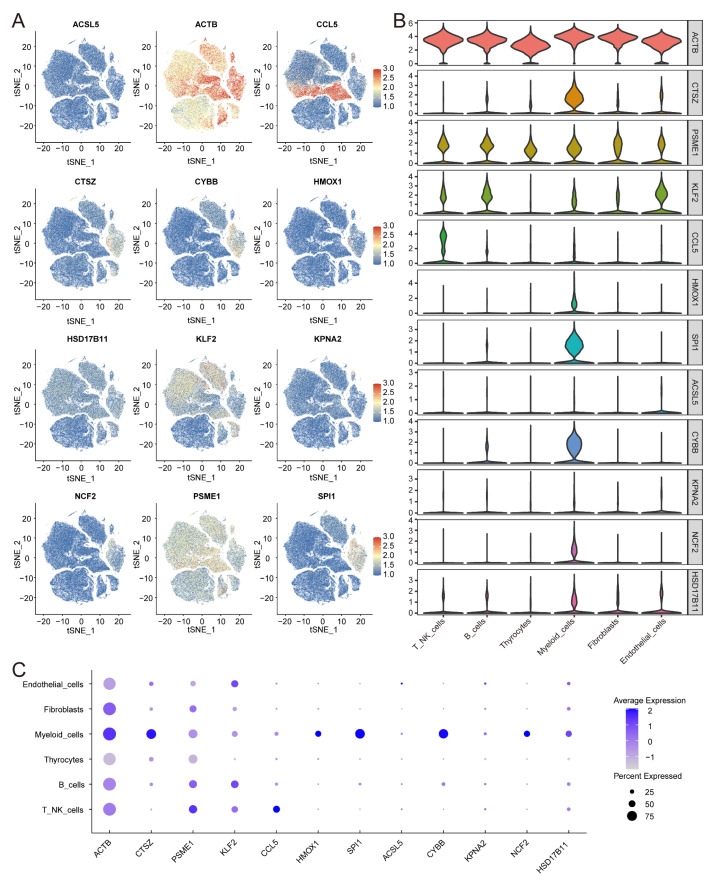
Validation of IRFGs in the scRNA-seq data and clinical samples: (**A**) Feature plot drawn for each gene independently using t-SNE. (**B**,**C**) The expression levels of each gene are depicted using violin and dot plots, categorised by cell type. *ACTB*, *CTSZ*, *SPI1*, *HMOX1*, *CYBB*, *KPNA2*, *NCF2*, and *HSD17B11* were expressed predominantly in myeloid cells; *ACSL5* and *KLF2* were expressed primarily in endothelial cells; and PSME1 was expressed mainly in T/NK cells.

To investigate the source and underlying mechanisms of ferroptosis-related genes in the TME, we analysed the expression of 12 genes across different cell types and visualised the results with feature plots (Figure 13A), violin plots (Figure 13B), and dot plots (Figure 13C). Among these genes, *ACTB*, *CTSZ*, *SPI1*, *HMOX1*, *CYBB*, *KPNA2*, *NCF2*, and *HSD17B11* were expressed predominantly in myeloid cells, *ACSL5* and *KLF2* were expressed primarily in endothelial cells, and *PSME1* was expressed mainly in T/NK cells.

### 3.11. Expression of IRFGs Genes in the Tumour Tissues

**Figure 14:**
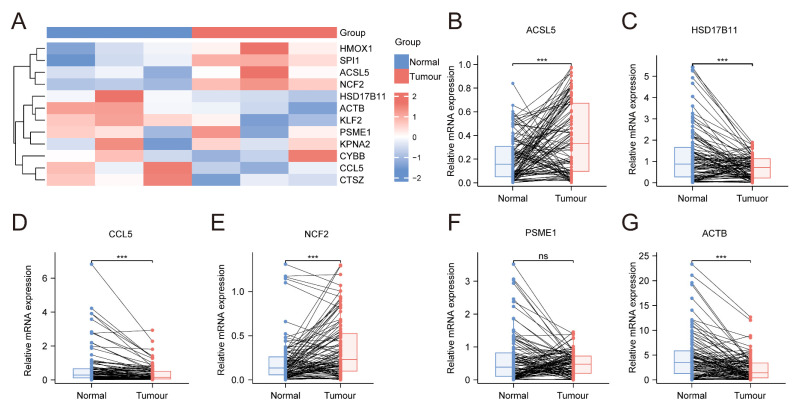
(**A**) Heatmap of RNA-seq data from thyroid tumour tissues (*n* = 3) and paired adjacent normal tissues, illustrating the expression profiles of IRFGs. (**B**–**G**) Thyroid tumour tissues (*n* = 100) and their paired adjacent normal tissues (*n* = 100) were used to validate the expression levels of IRFGs, including *ACSL5*, *HSD17B11*, *CCL5*, *NCF2*, *PSME1*, *ACTB* (*n* = 100), and paired adjacent normal tissues. *n* represents the number of patients involved in the analysis. ***: *p* < 0.001.

Using our previous RNA-seq data from a thyroid tumour and normal tissues, the expression levels of IRFGs were verified, and the results were consistent with the TCGA-THCA data (Figure 14A). Additionally, qRT–PCR was performed to validate the expression of IRFGs in tumour (*n* = 100) and paired adjacent normal tissues (Figure 14B–G). Compared to normal tissues, the expression levels of *ACSL5* and *NCF2* were increased, and those of *HSD17B11*, *CCL5*, and *ACTB* were decreased. The expression of PSME1 showed no significant difference. These findings are consistent with the results from the database, suggesting that these genes may be involved in the key pathogenesis of thyroid cancer.

## 4. Discussion

The incidence of thyroid cancer continues to rise annually, accompanied by high rates of lymph node metastasis and disease recurrence. This underscores the pressing need to identify reliable biomarkers that can enhance disease diagnosis and optimise treatment strategies. In the TME, genes associated with ferroptosis progression play important roles in regulating the tumour immunotherapy response and disease progression; however, few studies have investigated this topic in the context of thyroid cancer. In this study, we comprehensively analysed bulk and single-cell RNA-seq data to investigate IRFGs within the TME of thyroid cancer patients. A prognostic risk model was constructed based on the six IRFGs. Additionally, these genes may serve as potential targets to reshape the tumour immune microenvironment of thyroid cancer, which provides new perspectives for treatment and prognostic evaluation.

Ferroptosis is an iron-dependent form of programmed cell death. The ferroptosis metabolic pathway of immune cells has a major impact on the antitumour ability and immune escape of tumour cells in the TME [58,59]. Through comprehensive analysis, this study elucidated the expression patterns of 12 IRFGs in the TME of thyroid cancer, including *ACTB*, *CTSZ*, *PSME1*, *KLF2*, *CCL5*, *HMOX1*, *SPI1*, *ACSL5*, *CYBB*, *KPNA2*, *NCF2*, and *HSD17B11*. Our findings indicate that these genes not only contribute to tumour progression but also may regulate immune evasion [60]. Current research on thyroid cancer has focused primarily on the effects of agents that inhibit ferroptosis in vitro cellular experiments [61,62], while the impact of ferroptosis inhibitors on the tumour immune microenvironment of thyroid cancer remains to be explored. Studies have shown that IFN-γ mediated by CD8+ T cells can induce ferroptosis [24]. Our study further confirmed the immunosuppressive characteristics of the TME in high-risk patients, suggesting that ferroptosis may play a crucial regulatory role in the immune landscape within the TME. These findings provide new insights into expanding therapeutic strategies for thyroid cancer.

Several IRFGs identified in this study are significantly associated with immune infiltration characteristics in the TME. *ACSL5*, acyl-CoA synthetase long chain family member 5, converts free long-chain fatty acids to acyl-CoA esters [63], which are critical for lipid synthesis and fatty acid degradation. Zhang et al. [64] have shown that *ACSL5* was associated with ferroptosis and influenced the metabolic state of tumour cells. Therefore, targeting *ACSL5* may enhance the therapeutic efficacy of ferroptosis inducers and improve the sensitivity of thyroid cancer to anticancer drugs. *CCL5*, C-C motif chemokine ligand 5, is a key chemokine involved in the recruitment and activation of immune cells [65]. It can enhance the recruitment of NK cells and CD8+ T cells, improving the antitumour immune response [66,67]. Our study revealed that with decreased expression in thyroid cancer tissues, *CCL5* may be associated with immune escape [68,69]. Targeting this gene may increase immune cell infiltration at the tumour site, thereby improving the efficacy of immune checkpoint inhibitors [70]. *NCF2*, neutrophil cytosolic factor 2, is involved in the formation of the NADPH oxidase complex, which further generates reactive oxygen species in neutrophils [71]. *NCF2* is abnormally expressed in various cancers and is linked to macrophage infiltration and transformation in the TME [72,73]. *PSME1*, proteasome activator subunit 1, is associated with the production of antigenic peptides presented by MHC I molecules [74], and its expression level is associated with the treatment response to immune checkpoint inhibitors in patients with lung cancer [75]. Given the key role of *PSME1* in protein degradation and antigen presentation, its abnormal expression may affect the biological behaviour and immune evasion ability of thyroid cancer cells. Studies in recent years have shown that *ACTB* is associated with immune infiltration in cancers [76]. Consistent with the findings of Gu et al. [77], *ACTB* expression is reduced in thyroid cancer tissues. Its downregulation may influence the TME by altering immune cell recruitment, adhesion, and migration. *SPI1* is a member of the E26 transformation-specific transcription factor family, which not only regulates the differentiation of myeloid cells and B cells but also plays a crucial role in tumour immune evasion, particularly by regulating PD-L1 expression to promote CD8+ T cell exhaustion. Therefore, *SPI1* may serve as a novel therapeutic target for immunotherapy [78]. *KLF2* is a zinc-finger transcription factor that serves as a crucial regulator of both vascular and immune homeostasis. Its roles in maintaining cellular quiescence, suppressing excessive inflammation, and reversing immune cell trafficking underscore its potential as a therapeutic target for modulating inflammatory and immune responses in various pathological contexts [79].

Immunotherapy, particularly immune checkpoint inhibitors (ICIs), has demonstrated efficacy in certain types of advanced thyroid cancer. Studies have reported that combination therapies involving PD-1 inhibitors hold promise in thyroid cancer patients who are refractory to conventional treatments [80,81]. Additionally, ICAM-1-targeted CAR-T cell therapy may offer a potential treatment option for high-grade thyroid cancer [82]. However, a considerable proportion of patients exhibit no significant response to ICI monotherapy, highlighting the need for further investigations into resistance mechanisms and combination treatment strategies. Therapy targeting IRFGs may enhance the antitumour effects of immune cells, suppress the function of immunosuppressive cells, and improve immune resistance in thyroid cancer [83]. Although our findings indicate that they are crucially related to the prognosis of thyroid cancer patients, there are limited reports on the role of these genes in ferroptosis. The precise mechanisms of these IRFGs remain unclear. Our next step is to elucidate their specific roles in ferroptosis by modulating the expression of these genes. Research on IRFGs may pave the way for a shift in thyroid cancer treatment, moving beyond a one-size-fits-all approach toward a ferroptosis-driven immune microenvironment remodelling and immune reactivation-based combination immunotherapy strategy. This approach has the potential to increase the efficacy of immunotherapy and ultimately improve patient outcomes.

The expression profiles of IRFGs were used to classify the tumour samples, revealing distinct survival rates among different subtypes. This finding aligns with the well-documented differences in prognosis across various pathological types of thyroid cancer [84]. Studies have demonstrated that the elevated expression of GPX4, a core ferroptosis regulator, is associated with high-grade thyroid cancer [85], and another key regulator, SLC7A11, is linked to poor prognosis [86]. These findings suggest that thyroid cancers with different malignancy levels may exhibit varying degrees of ferroptosis dependency. In this study, we identified significant differences in the expression levels of certain IRFGs among different thyroid cancer subtypes, highlighting their potential as molecular targets for high-grade thyroid cancer. This discovery may provide new insights into personalised treatment strategies.

Using the IRFGs identified above, we trained and validated a prognostic model for thyroid cancer. Six key genes involved in immune-related ferroptosis, namely *ACSL5*, *HSD17B11*, *CCL5*, *NCF2*, *PSME1*, and *ACTB*, were further screened. Correlation analysis between these genes and the immune characteristics of the TME revealed that the abovementioned six genes were correlated with both the relative abundance of immune cell populations and immune response activity in thyroid cancer. In this model, the performance in the training set was well replicated in the validation set, demonstrating favorable AUC values and strong discriminative ability, indicating its generalizability and stability. Moreover, the high-risk group had a worse prognosis and greater aggressiveness, and the model also reflected the immune status of the TME. Although age is a non-negligible risk factor for the prognosis of thyroid cancer patients, the risk score in this model emerged as a significant prognostic risk factor. The predictive power of this model surpassed that of the existing clinical and pathological staging systems for thyroid cancer. Furthermore, the model results demonstrated a significant difference in MSI levels between the groups, suggesting that IRFGs may be associated with the genomic characteristics of thyroid cancer.

The genes identified in our prognostic model may serve as biomarkers for screening high-risk thyroid cancer patients and assessing their prognosis. In breast cancer research, a risk prediction model comprising ten ferroptosis-related genes has been developed [87], while in thyroid cancer research, a similar model consisting of eight ferroptosis-related genes has been constructed [88]. Compared with these models, our approach incorporates immune cell infiltration within the TME, making it more clinically relevant and potentially more cost-effective for prognostic assessment. However, due to the generally favorable prognosis of thyroid cancer, its low mortality rate, and its multifactorial influence on long-term survival, our model results in relatively lower long-term AUC values. To address this limitation, we plan to integrate larger independent clinical cohorts in future studies and comprehensively consider relevant clinical variables to further refine and enhance the model’s long-term predictive accuracy. With extensive clinical validation, our model may ultimately be applied in clinical practice to identify high-risk thyroid cancer patients, facilitating more proactive and personalised treatment strategies. For instance, patients identified as high-risk may benefit from ferroptosis inducers combined with immunotherapy, potentially improving overall survival outcomes.

Single-cell data were used to validate the source and expression levels of the IRFGs. After cluster annotation, the results of cell communication revealed that the communication between thyroid follicle cells and myeloid cells was the strongest. We subsequently confirmed the expression levels of the IRFGs within the clusters. The results showed that *ACTB*, *HSD17B11*, and *NCF2*, key genes in the risk prediction model, are expressed primarily in myeloid cells. Myeloid cells are critical for maintaining tissue homeostasis and mediating inflammation [89,90]. CSF-1R kinase inhibitors have been shown not only to reduce the immunosuppressive effects of macrophages but also to enhance CD8+ T cell infiltration within the TME [91,92]. Additionally, dendritic cells (DCs) play crucial roles in determining the effectiveness of immunotherapy and hold promising potential in the field of tumour vaccines [93,94]. Furthermore, inhibiting the function of myeloid-derived suppressor cells (MDSCs) can enhance the efficacy of NK cell-based immunotherapy [95]. In the thyroid cancer microenvironment, myeloid cells can be activated by ligands secreted by thyroid follicle cells, thus participating in immune surveillance. We speculate that the potential mechanism of IRFGs in thyroid cancer may be linked to their role in regulating the immunosuppressive function of myeloid cells. By modulating IRFG expression, it may be possible to alter myeloid cell activity, thereby attenuating their immunosuppressive effects within the TME and offering a promising strategy to increase therapeutic efficacy.

Although this study validated the differential expression of IRFGs in thyroid cancer through multi-omics data analysis and qRT–PCR experiments and explored their potential roles in ferroptosis regulation and TME remodelling, the current findings are still primarily based on bioinformatics analysis. Therefore, in future studies, we will conduct further experimental validation, including gene knockout and gene overexpression experiments, to elucidate the specific biological functions of these genes in ferroptosis induction and assess their feasibility as potential therapeutic targets. Due to the limited availability of publicly accessible thyroid cancer transcriptomic datasets with complete survival information, we were unable to validate our model in an independent external cohort. Moreover, since the prognostic model is primarily based on patient samples from public databases, there may be sample selection bias, highlighting the need for further evaluation of the model’s applicability in large-scale real-world clinical settings. To increase the clinical relevance and translational potential of this study, we plan to incorporate large, long-term independent clinical cohorts in future research. This will allow us to assess the model’s robustness in independent datasets and ensure its applicability across diverse patient populations. Furthermore, in the future, the model can be validated and calibrated through blood tests potentially applied for disease risk assessment in clinical patients with thyroid cancer.

## 5. Conclusions

This study integrated bulk transcriptomics, single-cell RNA sequencing, and multi-omics analyses from the TCGA and GEO database to identify 12 IRFGs whose expression is associated with the survival of thyroid cancer patients. Functional enrichment analysis suggested that IRFGs are involved in the core processes of ferroptosis by regulating lipid peroxidation and redox homeostasis imbalance. Additionally, these genes may contribute to an immunosuppressive microenvironment by recruiting regulatory T cells and inhibiting antigen presentation, potentially affecting patient responses to immunotherapy. A prognostic model comprising six key genes was constructed, demonstrating significant value in predicting patient outcomes. Further validation using qRT–PCR in 100 paired thyroid cancer and adjacent normal tissues revealed consistent upregulation of *ACSL5* and *NCF2* in tumour tissues and downregulation of *HSD17B11*, *CCL5*, and *ACTB*. These findings suggest that these IRFGs may serve as potential therapeutic targets, improving the efficacy of immunotherapy and optimising immunotherapeutic strategies.

## Data Availability

The original contributions presented in this study are included in the article/Supplementary Materials. Further inquiries can be directed to the corresponding author.

## Supplementary Materials

The following supporting information can be downloaded at: https://www.mdpi.com/article/10.3390/biomedicines13040903/s1, Table S1. Ferroptosis genes from WGCNA module. Table S2. Ferroptosis genes from GeneCards, FerrDb and the GSEA and MsigDB databases. Table S3. Immune response-related genes retrieved from the ImmPort database for analysis. Table S4. Primer sequences used in qRT-PCR for gene amplification. Table S5. Genes included in the MEgreen module Table S6. Ferroptosis-related genes with significant differential expression. Table S7. GO enrichment analysis for IRFGs. Table S8. Genes associated with prognosis identified through survival analysis. Table S9. Pearson correlation analysis of the relationships between the six IRFGs and immune cell populations using the CIBERSORTx tool. Table S10. Pearson correlation analysis of the relationships between the six IRFGs and immune response activity via ssGSEA. Table S11. Differential expression analysis between cell groups. Table S12. CellChat counts between cell groups. Table S13. CellChat weight between cell groups. Table S14. Communication probabilities between ligand-receptor pairs.
